# Investigation of CO_2_ Extract of *Portulaca oleracea* for Antioxidant Activity from Raw Material Cultivated in Kazakhstan

**DOI:** 10.1155/2022/6478977

**Published:** 2022-04-22

**Authors:** Meruyert I. Tleubayeva, Raisa M. Abdullabekova, Ubaidilla М. Datkhayev, Margarita Yu. Ishmuratova, Mereke B. Alimzhanova, Kaldanay K. Kozhanova, Aida M. Seitaliyeva, Kairat S. Zhakipbekov, Zhanar B. Iskakova, Elmira A. Serikbayeva, Elena V. Flisyuk

**Affiliations:** ^1^Department of Organization and Management and Economics of Pharmacy and Clinical Pharmacy, Department of Engineering Disciplines, School of Pharmacy, NJSC Karaganda Medical University on the Karaganda Medical University, Almaty 050000, Tole Bi 88 Str, Kazakhstan; ^2^Departament of Pharmaceutical Disciplines and Chemistry, NJSC Karaganda Medical University, Karaganda 100008, Gogol 40 Str, Kazakhstan; ^3^Department of Botany, Karaganda Buketov University, Karaganda 100024, Universitetskaya 28 Str, Kazakhstan; ^4^Department of Thermal Physics and Technical Physics, Faculty of Physics and Technology, Al-Farabi Kazakh National University, Almaty 050040, Al-Farabi Ave 71, Kazakhstan; ^5^Department of Fundamental Medicine Faculty of Medicine and Health, Al-Farabi Kazakh National University, Almaty 050040, Al-Farabi Ave 71, Kazakhstan; ^6^Research Institute of New Chemical Technologies, L.N. Gumilyov Eurasian National University, Nur-Sultan 010000, Satpaev 2 Str, Kazakhstan; ^7^Departament of Technology of Drugs Forms, St. Petersburg State Chemical and Pharmaceutical University, Petersburg 197376, Popov 14 Str, Kazakhstan

## Abstract

Medicinal plants remain as an important resource in the fight against many diseases, especially in developing countries. Antioxidants are substances capable of delaying, retarding, and preventing the oxidation of lipids or substances that delay or prevent free radical reactions during lipid oxidation. Natural antioxidants such as ascorbic acid, tocopherol, phenolic compounds, and flavonoids are a safe alternative to chemical antioxidants. In present work, results of antioxidant activity of raw materials from the cultivated plant *Portulaca oleracea* are presented. The extraction time was optimized to 780 minutes; the yield of extractive substances was 1.25% in the production of CO_2_ extract under subcritical conditions. For the first time, the antioxidant activity of *Portulaca oleracea* CO_2_ extract was determined by the amperometric method. Gas chromatography-mass spectrometry (GC-MS) chemical analysis of *Portulaca oleracea* CO_2_ extract dissolved in hexane revealed 37 components, including a complex mixture of aldehydes, alkanes, alkenes, esters, diterpenes, steroids, vitamin E, and carbohydrates. The investigation results showed that the *Portulaca oleracea* CO_2_ extract was promising for pharmaceutical, cosmetic, and food industries and had great potential for the prevention and treatment of diseases caused by oxidative stress.

## 1. Introduction

In the fight against diseases, medicinal plants still remain their importance and are a promising source of medicines, especially in developing countries [1]. About 3.4 billion of people use herbal medicines. Natural products were the integral parts of the ancient system of traditional medicine. According to the World Health Organization (WHO), medicinal plant is any plant which contains substances used for therapeutic purposes [2, 3].

Natural antioxidants are the interest for medicine among different biological activities. Antioxidants are important substances produced in the organisms to suppress oxidative stress. They can be divided into enzymatic and nonenzymatic ones. Oxidative stress is associated with a large number of lifestyle-related diseases, such as cardiovascular disease, cancer, diabetes, and aging. Excessive oxidative stress can lead to oxidation of biomolecules, which is accompanied by cell damage and intervenes in the pathogenesis of many human diseases [4].

Antioxidants are able to interact with free radicals to stop the chain reaction without damaging vital molecules [5]. These are compounds capable of delaying, retarding, and preventing the oxidation of lipids or substances that delay or prevent free radical reactions during lipid oxidation. Natural antioxidants such as ascorbic acid, tocopherol, phenolic compounds, and flavonoids are a safe alternative to chemical antioxidants [6, 7].

Currently, there are different methods for evaluating the antioxidant activity of compounds. So, electrochemical and spectrometric methods are widely used, as they are characterized by high sensitivity and the ability to analyze quickly. When using amperometric detection, compounds containing hydroxyl groups are well oxidized, and the detection level of polyphenols and flavonoids is 10^−9^–10^−12^ grams. The amperometric method allows measuring the amount of all antioxidants in the sample, which makes this investigation method more accurate [8–12].

It is noted that the therapeutic effect of extractive preparations does not depend on one active substance, but on the complex of all biologically active substances contained in it, enhancing, slowing down, or changing the type of action of basic substances [13].

Previous phytochemical studies [14–17] indicate that the medicinal plant *Portulaca oleracea* L. contains terpenoids, alkaloids, flavonoids, organic acids, minerals, and vitamins. This indicates the potential for antioxidant activity.

In our study, for the first time, the antioxidant activity of *P. oleracea* CO_2_ extract was determined by the amperometric method.

## 2. Materials and Methodology

### 2.1. Plant Material and *Portulaca oleracea* CO_2_ Extract

Raw material: the above-ground part of the cultivated plant *P. oleracea* was collected in 2-3 decades of August, 2020, in flowering phase. The place of collecting was N 42°52'07.8”; E 71°20'42.8” (Zhambyl region, Kazakhstan). The plant samples were identified by specialists of the Institute of Botany and Phytointroduction (Almaty city); typical specimen is stored in the herbarium fund of this institute.

Extraction: for the first time, CO_2_ extract from the above-ground part of the wild plants of *Portulaca oleracea* was obtained under precritical conditions, namely, pressure was 45–52 atmospheres, temperature was 19–22°C, extraction time was 540 minutes, and yield was 0.7%, as well as its component composition [18].

The extraction parameters of the cultivated plant *P. oleracea* were compared, which made it possible to recommend a change in the extraction time. Crushed air-dried raw materials (stems, leaves, and flowers) were extracted under pressure of 45–52 atmospheres, temperature of 19–22°C, extraction time of 780 minutes, and yield of 1.25%. The component composition was determined for the new extract.

### 2.2. The Determination of CO_2_ Extract Component Composition of *Portulaca oleracea* Cultivated Raw Material

CO_2_ extract of *Portulaca oleracea* and the different fractions from CO_2_ extract *of Portulaca oleracea* were injected using the 10 *μ*L Agilent syringe into the sample injection device of a Gas Chromatograph 7890A (Agilent, USA) coupled with a mass spectrometric detector 5975°C (Agilent, USA) in split mode- 10 : 1, injected sample volume- 1.0 *μ*l and inlet temperature- 250°C. Chromatography was performed using a DB-35MS capillary column with a length of 30 m, an inner diameter of 0.25 mm, and a film thickness of 0.25 *μ*m (Agilent, USA). The carrier gas Helium (>99.995%, Orenburg-Tehgas, Russia) was supplied at a constant rate of 1.0 mL min^−^1.

The temperature of the column thermostat was programmed from 50°С (holding time 1 min) to 270°С (holding time 15 min) with a heating rate of 5°С min^−^1. The analysis time was 60 minutes. The MSD interface temperature was 320°C, the temperature of the quadrupole was 180°C, and the ion source temperature was 230°C. It is detected in the ion-scanning mode in the range of mass numbers m/*z* 34–750 a.m.u.

Agilent MSD ChemStation software (version 1701EA) was used to control the gas chromatograph system and the system for recording and processing chromatographic data. The data processing included the determination of the retention times of the substance; peak areas as well as the processing of the spectral information obtained using the mass spectrometric detector. Mass spectra were identified applying the Wiley 11th edition and NIST'02 [18].

### 2.3. The Extraction of Biological Active Substances by Fractionation of *Portulaca oleracea* CO_2_ Extract

Fractions analysis of *P. oleracea* CO_2_ extract was carried out on a chromatograph. Sample preparation: fractionation was carried out on a silica gel column. Name of samples: 1st fraction - hexane, 2nd fraction - dichloromethane, 3rd fraction- ethyl acetate, 4th fraction - methanol. Gas chromatography with mass spectrometric detection (Agilent 7890 A/5975°C) was used as a method of analysis.

### 2.4. Evaluation of Antioxidant Activity of *Portulaca oleracea* CO_2_ Extract

The investigation was carried out on the basis of the technique for measuring the total content of fat-soluble antioxidants in food [19], drinks and food products, dietary supplements, and extracts of medicinal plants [20] by the amperometric method using the “Tsvet Yauza 01-AA” developed by “Khimavtomatika,” the scientific and production association (Moscow, Russia). Gallic acid was the standard for fat-soluble antioxidants; the range of determination was 0.00125–25 mg of gallic acid/*g* (%0). Acetone acidified with phosphoric acid was used as an eluent. Eluent preparation and calibration of gallic acid solutions with a mass concentration of 0.1, 0.2, 0.4, 1.0, and 2.0 mg/dm^3^ were carried out according to the certified method.

Quercetin served as a standard for water-soluble antioxidants; the range of determination was 0.2–4000 mg of quercetin/dm^3^. 70% ethyl alcohol was used as an eluent. Preparation of eluent and calibration solutions of quercetin with a mass concentration of 0.2, 0.5, 1.0, 2.0, and 4.0 mg/dm^3^ were carried out according to the certified method.

### 2.5. Method for Determining Antioxidant Activity by the FRAP Method

0.25 ml of 0.2 M phosphate buffer (pH = 6.6) and 0.25 ml of 1% solution of potassium hexacyanoferrate (III) were added to 0.1 ml of the test substances in the concentration range of 0.25, 0.5, 0.75, and 1.0 mg/ml [21, 22].

The reaction mixture was incubated for 20 minutes at 50°C; the reaction was stopped by adding 0.25 ml of 10% trichloroacetic acid solution. The mixture was centrifuged for 10 minutes (3000 rpm). The upper layer with a volume of 0.5 ml was mixed with 0.5 ml of distilled water and 0.1 ml of 0.1% FeCl_3_. The optical density was measured at 700 nm. The antioxidant activity (AOA) of the samples was compared with the AOA of ascorbic acid (AA).

Dilution was made at the rate of 1 mg of substance per 1 ml of solvent. Each sample was tested in three parallel runs. It was carried out at a temperature of 20 ± 2°C, natural light period.

## 3. Result and Discussion

### 3.1. Determination of Component Composition of CO_2_ Extract of Cultivated *P. oleracea*

Chemical analysis of *P. oleracea* CO_2_ extract dissolved in hexane was carried out by the GC-MS method. Thirty-seven components were identified, including complex mixture of aldehydes, alkanes, alkenes, esters, diterpenes, steroids, vitamin E, and carbohydrates (Table 1).

### 3.2. Isolation of Biologically Active Compounds by Fractionation of the *Portulaca olеracea* CO_2_ Extract

**Figure 1:**
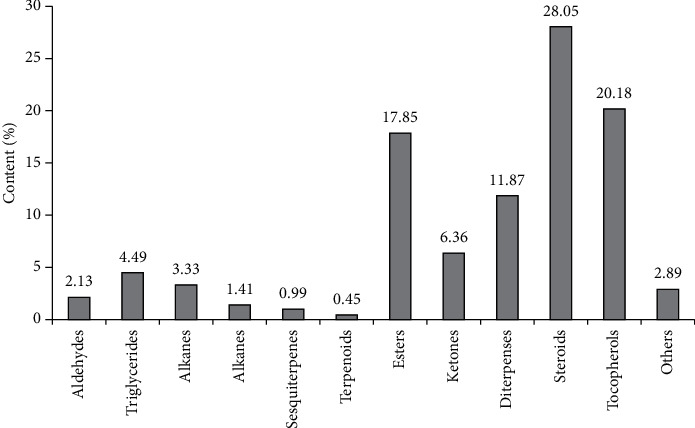
The ratio of the groups of biologically active substances of the hexane fraction of the *Portulaca olеracea* CO_2_ extract.

Chromatographic analysis of the hexane fraction of the *Portulaca оlеracea* CO_2_ extract showed the presence of 48 components, which included aldehydes (2.13%), triglycerides (4.49%), alkanes (3.33%), alkenes (1.41%), sesquiterpenes (0.99%), terpenoids (0.45%), esters (17.85%), ketone (6.36%), diterpenes (11.87%), steroids (28.05%), *γ*-tocopherols (2.56%), vitamin E (17.62%), and others (2.89%) (Table 2, Figure 1).

**Figure 2:**
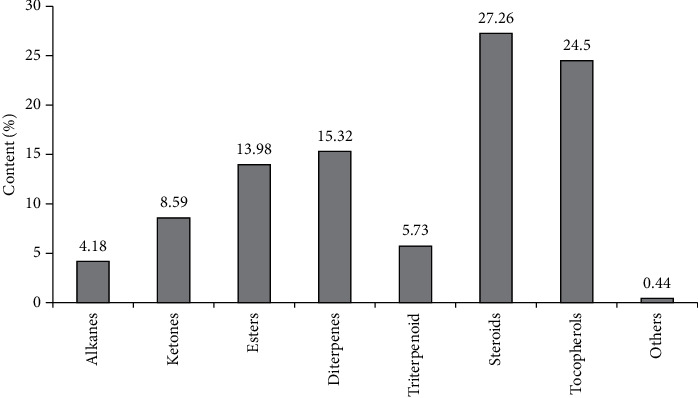
The ratio of the main groups of biologically active substances of the *Portulaca oleracea* dichloromethane fraction.

Chromatographic analysis of the dichloromethane fraction of the *Portulaca оlеracea* CO_2_ extract showed the presence of 18 components, including alkanes (4.18%), ketone (8.59%), esters (13.98%), diterpenes (15.32%), triterpenoid (5.73%), steroids (27.26%), *γ*-tocopherols (3.28%), vitamin E (21.22%), and others (0.44%) (Table 3, Figure 2).

**Figure 3:**
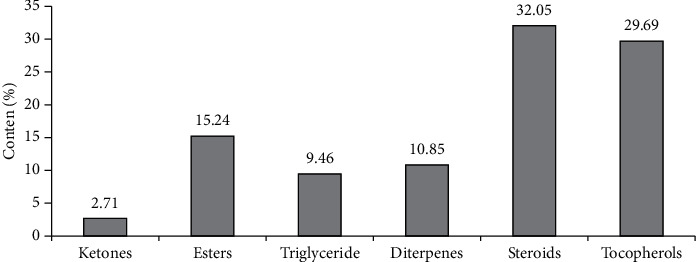
The ratio of the main groups of biologically active compounds of the *Portulaca oleracea* ethyl acetate fraction.

Chromatographic analysis of the ethyl acetate fraction of the *Portulaca olеracea* CO_2_ extract contains 10 components, including ketone (2.71%), esters (15.24%), triglyceride (9.46%), diterpenes (10.85%), steroids (32.05%), d1-*α*-tocopherols (29.69%) (Table 4, Figure 3).

**Figure 4:**
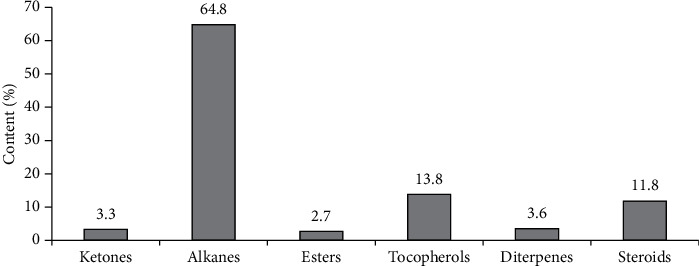
The ratio of the main groups of biologically active compounds of the *Portulaca oleracea* methanol fraction.

Chromatographic analysis of the methanol fraction of the *Portulaca оlеracea* CO_2_ extract revealed 8 components, namely, ketone (3.3%), alkanes (64.8%), esters (2.7%), vitamin E (13.8%), diterpenes (3.6%), and steroids (11.8%) (Table 5), (Figure 4).

### 3.3. Antioxidant Activity of *Portulaca оlеracea* CO_2_ Extract

#### 3.3.1. Amperometric Method

Determination of the antioxidants sum composition by the amperometric method is based on measuring the electric current caused by oxidation of the antioxidant molecule on the surface of the working electrode at a certain potential, which is converted into a digital signal. Output signals are displayed on the computer screen as peaks. The magnitude of the electric current depends on the nature and concentration of the test substance, the type and material of the working electrode, and the potential applied to the electrode.

The content of antioxidants in the studied samples of the *Portulaca оlеracea* CO_2_ extract was calculated in units of the quercetin and gallic acid concentration. The content of water-soluble antioxidants is 35.5385 ± 0.1457 mg/g, and the content of fat-soluble antioxidants is 34.8361 ± 0.0488 mg/g.

The reliability of the correlation coefficient was determined for water-soluble antioxidants *r*_xy_ = + 0.998, *p*> 99.9% and for fat-soluble antioxidants *r*_xy_ = + 0.994, *p*>99.9%. The relationship between concentration and peak area is direct, strong, and reliable, which indicates a high reliability of the approximation.

#### 3.3.2. FRAP Method (Ferric Reducing Antioxidant Power Assay)

The FRAP method (ferric reducing antioxidant power assay) is based on the reduction of Fe^3+^ ions to Fe^2+^ by antioxidants. The reduction reaction of K_3_[Fe(CN)_6_] with antioxidants is used, which is accompanied by the formation of a yellow-colored compound, namely, K_4_[Fe(CN)_6_]. The measurements are based on the ability of antioxidants to suppress the oxidative effect of reaction particles generated in the reaction mixture. Ascorbic acid was used as a reference drug. Samples were tested at concentrations of 0.25, 0.5, 0.75, and 1 mg/ml (Table 6).

Based on the data analysis, it can be seen that the *Portulaca oleracea* CO_2_ extract at concentrations from 0.25 to 1 mg/ml has a low antioxidant activity compared to the standard solution of ascorbic acid.

Earlier, 41–66 components were found, when studying the component composition of the *Portulaca oleracea* carbon dioxide extract from the raw material of a wild plant. Triterpenoids 6.62%–30.72%, tocopherols 1.46–3.41%, fatty acids 11.31–34.11%, and terpenoids 3.22%–7.07% made up the sum of the main compounds of chromatographic analysis by classes [18]. The component composition of the *Portulaca oleracea* carbon dioxide extract from the raw material of the cultivated plant included 37 components. The most known pharmacologically bioactive compounds of the therapeutic value are given in Tables 7and 8.

Antioxidant properties of *Portulaca oleracea* are associated with biologically active substances such as gallotannin, omega-3 fatty acids, ascorbic acid, tocopherol, kaempferol, quercetin, and apigenin [29, 30].

Using a simple, fast, and affordable single cell electrophoresis method to measure DNA fiber breakage, the results showed that an aqueous extract of *Portulaca oleracea*, in contrast to ethanol extract, had a high ability to reduce oxidative damage caused by high levels of fat by modulating the activity of antioxidant enzymes [14].

The antioxidant activity of the *Portulaca oleracea* medicinal plant material was determined at the plant maturation stages. It was determined by the reduction of 1,1-diphenyl-2-picrylhydrazyl (DPPH), by reducing properties of iron extracts (FRAP) and by the amount of ascorbic acid. On the basis of the obtained results, it was concluded that the total phenol content and antioxidant activity in mature *Portulaca oleracea* plants were higher than in immature plant stages [31].

The antioxidant activity of methanol extracts of various parts of the *Portulaca oleracea* and *Portulaca grandiflora* plants was studied using DPPH. For the first time, it was found that the *Portulaca oleracea* root had effective antioxidant activity [32].

According to Naciye Erkan, using TBARS analysis, it was shown that the fraction of *Portulaca oleracea* extract with the highest total quantitative content of phenolic compounds had antioxidant activity with the highest rate of lipid peroxidation suppression [33]. The antioxidant activity of aqueous and ethanol extracts of stems and leaves of the Tunisian species of *Portulaca oleracea* was determined using free radical discoloration ABTS, the reducing properties of Fe^3+^ extracts, and phosphomolybdenum analysis [34].

There were carried out the study of the antioxidant activity of methanol, ethanol, and aqueous extracts of *Portulaca oleracea* using such methods as FRAP [31, 34], DPPH [31, 32], phosphomolybdenum [34], TBARS [33], and single cell electrophoresis [14].

The study of the antioxidant activity of extracts from plant materials is of interest to scientists. The authors Syeda A.M. and Riazunnisa K. report on the determination by gas chromatography-mass spectrometry (GC-MS) of the component composition of aqueous and methanolic extracts of Madagascar periwinkle (*Catharanthus roseus*) and drumstick tree (*Moringa oleifera*) and found their antioxidant activity [35]. On the basis of plant extracts, a technology of multifunctional film has been developed [36, 37], which has the prospect of application in the pharmaceutical and food industries.

## 4. Conclusions

For the first time, there has been revealed the antioxidant activity of *Portulaca оlеracea* CO_2_ extract from raw materials cultivated in Kazakhstan. The results of the study included determination of the composition of the sum of antioxidants in the *Portulaca оlеracea* CO_2_ extract by the amperometric method. The composition concentration of the combination of fat-soluble and water-soluble antioxidants was established. The investigation results of antioxidant activity by the FRAP method allow us to conclude that the *Portulaca oleracea* CO_2_ extract in concentrations of 0.25–1 mg/ml has an antioxidant activity, which turned out to be lower than that of ascorbic acid, but promising for the pharmaceutical, cosmetic, and food industries.

## Figures and Tables

**Table 1:** The results of chromatographic analysis of *Portulaca olеracea* CO_2_ extract.

No.	Retention time (min)	Compound	Identification probability (%)	Percentage (%)	Groups of biological active compounds
1	11.0	4-Cyclopentene-1, 3-dione	84	0.79	Ketone
2	12.6	4-Hydroxy-butanoic acid	93	0.46	Butyrolactone
3	12.8	1-Butene, 4-isothiocyanato-	85	0.32	Isothiocyanic acid, 3-butenyl ester
4	13.5	1-(1'-Pyrrolidinyl)-2-propanone	95	0.99	Ketone
5	14.6	1-Amino-2,6-dimethylpiperidine	67	0.27	Piperidine derivative
6	15.8	2,5-Dimethyl-4-hydroxy-3(2H)-furanone	77	0.26	Ketone
7	16.9	4H-Pyran-4-one, 2,3-dihydro-3, 5-dihydroxy-6-methyl-	90	2.44	Pyran derivative
8	17.5	Benzyl nitrile	93	1.21	Benzene derivatives
9	18.5	Benzofuran, 2,3-dihydro-1-	80	1.06	Benzofuran derivatives
10	20.4	2-Methoxy-4-vinylphenol	93	2.07	Phenol
11	20.7	Pyrazine, 2-ethyl-5-methyl-	85	0.96	Pyrazine derivative
12	22.8	Pyrrolidine, 1-(1-cyclohexen-1-yl)-	71	0.94	Pyrrolidine derivative
13	23.2	4-(2, 6, 6-Trimethylcyclohexa-1, 3-dienyl)but-3-en-2-one	78	0.39	Steroid
14	25.1	Sucrose	72	10.85	Carbohydrate
15	26.0	Phosphonofluoridic acid, (1-methylethyl)-, cyclohexyl ester	70	1.43	Ester
16	26.2	3', 5'-Dimethoxyacetophenone	76	1.85	Ketone
17	26.9	Megastigmatrienone	75	0.21	Steroid
18	27.4	3, 7, 11, 15-Tetramethyl-2-hexadecen-1-ol	80	1.31	Alcohol
19	29.0	Imidazolo [1,2-a] pyrimidine-2,5(1H,3H)-dione, 3,7-dimethyl-	65	0.36	Ketone
20	30.6	4-((1E)-3-Hydroxy-1-propenyl)-2-methoxyphenol	82	0.76	Phenol
21	31.2	Benzoic acid, 4-hydroxy-3, 5-dimethoxy-, hydrazide	91	2.21	Heterocyclic compound
22	31.5	Phthalic acid, isobutyl octadecyl ester	64	3.03	Ester
23	32.5	Octahydro-2(1H)-quinolinone	66	1.70	Ketone
24	33.8	Phytol	76	5.80	Diterpene
25	34.1	Linoleic acid ethyl ester	72	0.69	Ester
26	35.6	Desulphosinigrin	70	16.96	Carbohydrate
27	36.6	5, 10-Diethoxy-2, 3, 7, 8-tetrahydro-1H,6H-dipyrrolo[1,2-a:1',2'-d]pyrazine	71	0.47	Pyrazine derivative
28	39.0	Heneicosane	87	0.81	Alkane
29	40.6	Heptacosane	80	0.68	Alkane
30	41.4	2-Methylhexacosane	84	1.02	Alkane
31	42.1	Hexacosane	93	20.67	Alkane
32	43.6	Octacosane	91	1.85	Alkane
33	46.4	Tetratetracontane	79	1.31	Alkane
34	48.7	Octacosanol	74	2.67	Alcohol
35	50.9	Vitamin E	95	6.42	Derivative of tocol
36	54.7	*β*-Sitosterol	90	3.78	Steroid
37	55.3	Phytol, acetate	70	0.96	Ester

**Table 2:** Chromatographic analysis of the hexane fraction of *Portulaca olеracea* CO_2_ extract.

No.	Retention time (min)	Compound	Identification probability (%)	Percentage (%)
1	12.7	2, 4-Heptadienal	89	0.96
2	14.4	Nonanal	91	0.22
3	15.8	2, 5-Furandione, 3-(1,1-dimethylethyl)-	88	0.27
4	16.1	Cyclohexanol, 1-methyl-4-(1-methylethyl)-	83	0.54
5	16.3	Octanoic acid	90	0.92
6	16.4	Cyclodecene	73	0.24
7	18.2	Nonanoic acid	87	1.45
8	18.7	Tetradecane	85	0.31
9	19.1	2, 4-Decadienal	87	0.67
10	19.4	Alfa-copaene	93	0.26
11	20.0	Decanoic acid	72	1.44
12	20.6	Caryophyllene	93	0.43
13	21.0	Undecanoic acid	84	0.40
14	21.9	5,9-Undecadien-2-one, 6,10-dimethyl-	85	0.38
15	22.5	Hexadecane	90	0.33
16	22.8	*γ*-Muurolene	73	0.30
17	23.0	trans-*β*-Ionone	90	0.28
18	23.2	3-Buten-2-one, 4-(2,2,6-trimethyl-7-oxabicyclo[4.1.0]hept-1-yl)-	74	0.24
19	23.2	Vanillin	75	0.19
20	24.0	Nonanoic acid, 9-oxo-, ethyl ester	85	0.31
21	24.4	Heptadecane	82	0.36
22	25.6	Tridecanoic acid	83	0.28
23	26.0	2(4H)-Benzofuranone, 5, 6, 7, 7a-tetrahydro-4,4,7a-trimethyl-, (R)-	74	0.82
24	26.1	Hexadecane, 2,6,10,14-tetramethyl-	69	0.15
25	26.3	Octadecane	89	0.25
26	26.5	Hexadecanal	82	0.28
27	27.4	3,7,11,15-Tetramethyl-2-hexadecen-1-ol	84	0.45
28	28.3	Nonadecane	89	0.34
29	28.6	2-Pentadecanone, 6, 10, 14-trimethyl-	93	2.87
30	29.9	Benzoic acid, undecyl ester	66	0.30
31	30.3	Hexadecanoic acid, methyl ester	75	0.39
32	30.7	1,4-Naphthalenedione, 2, 3, 6-trimethyl-	71	0.50
33	31.1	5, 9, 13-Pentadecatrien-2-one, 6, 10, 14-trimethyl-, (E,E)-	87	0.53
34	31.2	Benzoic acid, 4-hydroxy-3, 5-dimethoxy-, hydrazide	86	0.35
35	33.9	Phytol	94	11.87
36	34.4	*p*-Octylacetophenone	68	1.96
37	35.1	Ethyl oleate	91	5.73
38	38.6	Methyl 19-methyl-eicosanoate	68	2.69
39	39.3	4,8,12,16-Tetramethylheptadecan-4-olide	86	1.53
40	41.8	Docosanoic acid, ethyl ester	75	0.97
41	42.6	13-Methylheptacosane	74	1.59
42	49.9	*γ*-Tocopherol	60	2.56
43	51.0	Vitamin E	93	17.62
44	52.0	Phytol, acetate	84	2.83
45	53.2	Campesterol	91	4.54
46	53.7	Stigmasterol	83	2.86
47	54.9	*γ*-Sitosterol	91	20.65
48	55.4	Phytol, acetate	85	4.63

**Table 3:** Chromatographic analysis of the dichloromethane fraction of the *Portulaca olеracea* CO_2_ extract.

No.	Retention time (min)	Compound	Identification probability (%)	Percentage (%)
1	26.0	2(4H)-Benzofuranone, 5,6,7,7a-tetrahydro-4,4,7a-trimethyl-	78	1.00
2	27.4	3,7,11,15-Tetramethyl-2-hexadecen-1-ol	81	0.44
3	27.7	Tetradecanoic acid, ethyl ester	89	0.99
4	28.5	3-Buten-2-one, 4-(4-hydroxy-2,2,6-trimethyl-7-oxabicyclo[4.1.0]hept-1-yl)-	73	0.77
5	28.6	2-Pentadecanone, 6,10,14-trimethyl-	92	2.33
6	33.8	Phytol	94	15.32
7	34.3	*p*-Octylacetophenone	67	2.52
8	35.0	Ethyl oleate	88	7.15
9	38.1	Hexadecanoic acid, 1-(hydroxymethyl)-1,2-ethanediyl ester	65	0.62
10	38.6	Methyl 19-methyl-eicosanoate	68	1.36
11	39.2	4,8,12,16-Tetramethylheptadecan-4-olide	90	1.97
12	43.2	Hexacosane, 9-octyl-	80	4.18
13	45.2	Squalene	86	5.73
14	49.9	*γ*-Tocopherol	66	3.28
15	50.9	Vitamin E	95	21.22
16	53.2	Campesterol	84	4.51
17	54.7	*γ*-Sitosterol	92	22.75
18	55.3	Phytol, acetate	75	3.86

**Table 4:** Chromatographic analysis of the ethyl acetate fraction of the *Portulaca оlеracea* CO_2_ extract.

No.	Retention time (min)	Compound	Identification probability (%)	Percentage (%)
1	28.61	2-Pentadecanone, 6,10,14-trimethyl-	89	2.71
2	29.90	Benzoic acid, hept-2-yl ester	64	1.90
3	30.36	Benzoic acid, pentadecyl ester	78	4.05
4	31.43	Hexadecanoic acid	85	9.46
5	31.85	Methyl 8,11,14-heptadecatrienoate	82	2.15
6	33.76	Phytol	67	10.85
7	34.99	Ethyl oleate	88	7.14
8	50.92	DL-*α*-Tocopherol	86	29.69
9	53.17	Campesterol	74	5.32
10	54.68	*γ*-Sitosterol	88	26.73

**Table 5:** Chromatographic analysis of the methanol fraction of the *Portulaca оlеracea* CO_2_ extract.

No.	Retention time (min)	Compound	Identification probability (%)	Percentage (%)
1	33.8	Phytol	85	3.6
2	35.0	Ethyl oleate	71	2.7
3	39.0	Hexacosane	84	4.1
4	41.4	Octadecane, 3-ethyl-5-(2-ethylbutyl)-	65	3.3
5	45.0	Octacosane	92	53.4
6	48.7	Hentriacontane	80	7.3
7	50.9	Vitamin E	89	13.8
8	54.7	*γ*-Sitosterol	80	11.8

**Table 6:** Change in the optical density of solutions depending on the working solutions concentration.

No.	Samples	Optical density value at concentration (mg/ml)			
		0.25	0.5	0.75	1.0
1	Ascorbic acid (AA)	1.5539	1.5928	1.6775	1.7738
2	CO_2_ extract of *Portulaca oleracea* (CO_2_*P. oleracea*)	0.1314	0.2659	0.3878	0.7519

**Table 7:** Pharmacologically active compounds of the therapeutic value from the *Portulaca оlеracea* CO_2_ extract.

No.	Compound	Percentage (%)	Pharmacologic effect
1	4H-Pyran-4-one, 2,3-dihydro-3,5-dihydroxy-6-methyl-	2.44	Antidiabetic, antioxidant, antibacterial, anti-inflammatory, antifungal activity [23], and anticancer activities [24]
2	Benzofuran, 2,3-dihydro-	1.06	Anti-HIV, anticancer, antibacterial, and antifungal activities [24]
3	2-Methoxy-4-vinylphenol	2.07	Antibacterial, anti-inflammatory [23], antioxidant, and anticancer activities [23, 24]
4	Desulphosinigrin	16.96	Antioxidant activity [24]
5	Vitamin E	6.42	Antioxidant and anti-inflammatory activities [24]

**Table 8:** Pharmacologically active compounds of therapeutic value (hexane, dichloromethane, ethyl acetate, and methanol fraction) of *Portulaca оlеracea* CO_2_ extract.

No.	Compound	Hexane fraction		Dichloromethane fraction		Ethyl acetate fraction		Methanol fraction		Pharmacologic effect
		Percentage (%)	The presence or absence of an ingredient	Percentage (%)	The presence or absence of an ingredient	Percentage (%)	The presence or absence of an ingredient	Percentage (%)	The presence or absence of an ingredient	
1	Vitamin E	17.62	+	21.22	+	—	—	13.8	+	Antioxidant and anti-inflammatory activities [24].
2	Decanoic acid	1.44	+	−	−	−	−	−	−	Triglyceride. Antibacterial, anti-inflammatory, anticancer, and antioxidant activities [23].
3	2-Pentadecanone, 6,10,14-trimethyl	2.87	+	5.33	+	2.71	+	−	−	Antidiabetic potential and moderate anticholine esterase activities [25].
4	*γ*-Tocopherol	2.56	+	3.28	+	−	−	−	−	Anti-inflammatory property [24].
5	Campesterol	4.54	+	4.51	+	5.32	+	−	−	Phytosterols. Sterols have the ability to lower cholesterol levels. It is also effective in cancer prevention [26].
6	Stigmasterol	2.86	+	−	−	−	−	−	−	Antimicrobial and antioxidant activities [24]. Clinosterol is a class of phytosterols, a triterpenoid. Sterols have the ability to lower cholesterol levels. It is also effective in preventing cancer [26].
7	*γ*-Sitosterol	20.65	+	22.75	+	26.73	+	11.8	+	Phytosterols. Sterols have the ability to lower cholesterol levels. It is also effective in cancer prevention [26].
8	2(4H)-Benzofuranone, 5,6,7,7a-tetrahydro-4,4,7a-trimethyl-	—	—	1.00	+	—	—	—	—	Benzofuran derivatives have biological activity as an antidepressant, antitumor, antiviral, antifungal, antioxidant, antipsychotic agent [27].
9	Squalene	−	−	5.73	+	−	−	−	−	Triterpenoid. Antioxidant, hypolipidemic, and antitoxic effects [28].
10	Hexadecanoic acid 9,46	−	−	−	−	9.46	+	−	−	Triglyceride. Antitumor and antihelmintic properties [23]
11	DL-*α*-Tocopherol	−	−	−	−	29.69	+	−	−	Antioxidant and anti-inflammatory activities [24]

−, absence of ingredient; +, presence of ingredient.

## Data Availability

The datasets used and/or analyzed during the current study are available from the corresponding author upon request.
